# Evidence of Polyphenols Efficacy against Dry Eye Disease

**DOI:** 10.3390/antiox10020190

**Published:** 2021-01-28

**Authors:** Gaia Favero, Enrico Moretti, Kristína Krajčíková, Vladimíra Tomečková, Rita Rezzani

**Affiliations:** 1 Anatomy and Physiopathology Division, Department of Clinical and Experimental Sciences, University of Brescia, 25123 Brescia, Italy; gaia.favero@unibs.it (G.F.); enrico.moretti@unibs.it (E.M.); 2 Department of Medical and Clinical Biochemistry, Faculty of Medicine, Pavol Jozef Šafárik University, 040 12 Košice, Slovakia; kristina.ugrayova@gmail.com (K.K.); vladatomeckova@yahoo.com (V.T.); 3 Interdipartimental University Center of Research “Adaption and Regeneration of Tissues and Organs (ARTO)”, University of Brescia, 25123 Brescia, Italy

**Keywords:** antioxidants, dry eye disease, epithelial corneal cells, inflammation, polyphenols

## Abstract

Dry eye disease is a multifactorial pathology compromising the quality of life of patients, resulting in significant damage of the ocular surface and discomfort. The current therapeutical strategies are not able to definitively resolve the underlying causes and stop the symptoms. Polyphenols are promising natural molecules that are receiving increasing attention for their activity/effects in counteracting the main pathologic mechanisms of dry eye disease and reducing its symptoms. In the present review, a deep literature search focusing on the main polyphenols tested against dry eye disease was conducted, analyzing related in vitro, in vivo, and clinical studies to provide a comprehensive and current review on the state of the art. Polyphenols present multiple effects against dry eye diseases-related ocular surface injury. In particular, the observed beneficial effects of polyphenols on corneal cells are the reduction of the pathological processes of inflammation, oxidative stress, and apoptosis and modulation of the tear film. Due to numerous studies reporting that polyphenols are effective and safe for treating the pathological mechanisms of this ocular surface disease, we believe that future studies should confirm and extend the evidence of polyphenols efficacy in clinical practice against dry eye disease and help to develop new ophthalmic drug(s).

## 1. Introduction

The definition of dry eye disease (DED) was redefined several times during the last decades. In 1995, the National Eye Institute stated that “Dry eye is a disorder of the tear film, due to tear deficiency or excessive evaporation, which causes damage to the interpalpebral ocular surface and is associated with ocular discomfort” [1]. In 2007, the Tear Film and Ocular Surface Society restored the definition in “Dry eye is a multifactorial disease of the tears and ocular surface that results in symptoms of discomfort, visual disturbance and tear film instability with potential damage to the ocular surface. It is accompanied by increased osmolarity of the tear film and inflammation of the ocular surface” [2]. In 2017, the same Society redefined further DED as “a multifactorial disease of the ocular surface characterized by a loss of homeostasis of the tear film and accompanied by ocular symptoms, in which tear film instability and hyperosmolarity, ocular surface inflammation and damage, and neurosensory abnormalities play etiological roles” [3]. Data from big observational studies show that prevalence of DED varies greatly among the countries: In the United States, it reaches 6.8% of the population [4], while in India, DED affects 32% of the population [5]. In France, Germany, Italy, Spain, Sweden, and the United Kingdom, the estimated prevalence of DED among patients reporting to ophthalmologists was less than 0.1% [6]. Eyelid anatomy seems potentially implicated in the ethnic differences in the development of DED [7]. It is important to underline that the prevalence of DED is influenced by geographic location, climatic conditions, and patient’s lifestyle, but notably it even depends also on whether the data were generated from patients presenting at optometry practices/eye hospitals or obtained from general population [5,6]. Though, to date, there is abundant data on DED in adults, minimal literature exists on this ocular surface disease in the pediatric population.

### 1.1. Pathophysiology of Dry Eye Diseases

The risk factors of DED include age [8], female sex (affecting especially postmenopausal women and women with hormonal replacement therapy [9,10]), autoimmune diseases [11], various types of systemic drugs [12], mood disorders [13], contact lenses use [14], vitamin A and D deficiencies, and a few other conditions [15,16,17]. Women present a prevalence of DED between 50% and 70% higher than men, which is in large part attributable to the effects of sex steroids as well as to sex-specific autosomal factors, although such differences become more significant with age [17,18,19]. The interaction effect between sex and pain sensitivity is another element which plays a relevant role in this disease, in particular, DED symptoms gain in women is strictly correlated with remarkable pain sensitivity, which may be a possible reason of greater DED prevalence in women [20]. Prevalence of DED has also increased with the modern office environment that involves intense computer use and air conditioning, factors that desiccate the eyes [21]. All these determinants lead either to reduced secretion of tear fluid defined aqueous-deficient dry eye or higher tear fluid evaporation recognized as evaporative dry eye. These categories are not mutually exclusive and many patients manifest a combination of both these DED pathogenic mechanisms. Both variations have in common tear hyperosmolarity which leads to the downward spiral of DED pathophysiological processes: activation of corneal epithelium’s mitogen-activated protein kinase (MAPK) p38 and c-Jun N-terminal kinase (JNK), nuclear factor-kB (NF-kB), and activator protein-1 (AP-1) which are responsible for pro-inflammatory interleukins, matrix metalloproteinases (MMPs), and other chemokines production [8,22]. The core of DED etiology is a vicious cycle, where tear film instability and hyperosmolarity trigger an inflammatory cascade which lead to ocular surface damage and further loss of tear film homeostasis [23]. Tear film instability is at the basis of DED symptoms and, hence, a starting point for inflammation. Tear film hyperosmolarity is linked to cornea and conjunctiva damage, so altering epithelial cells and stimulating resident inflammatory cells [24,25]. In turn, corneal and conjunctival stress stimulates the neurosensory arc, inducing neurogenic lacrimal gland inflammation and altering tear fluid. Furthermore, inflammation, related to an imbalance of ocular homeostasis, is mediated by immune cells [26]. Immune cells release inflammatory cytokines that, in turn, degrade the corneal epithelial barrier and promote apoptosis in the conjunctiva and lacrimal gland. Thus, such processes alter the morphology of the ocular surface, accelerate DED progression, and promote symptoms onset [26,27].

Hyperosmolar conditions, inflammatory and matrix-remodeling factors stimulate ocular oxidative stress. These pathogenic factors are also related to the production of reactive oxygen species (ROS), to the decrement of antioxidant enzymes, such as glutathione peroxidase-1 and superoxide dismutase1 (SOD1), and to the increment of heme oxygenase-1 and cycloxygenase2 activity, thus disrupting the physiological balance between antioxidative enzymes and ROS [28,29,30,31,32].

**Figure 1:**
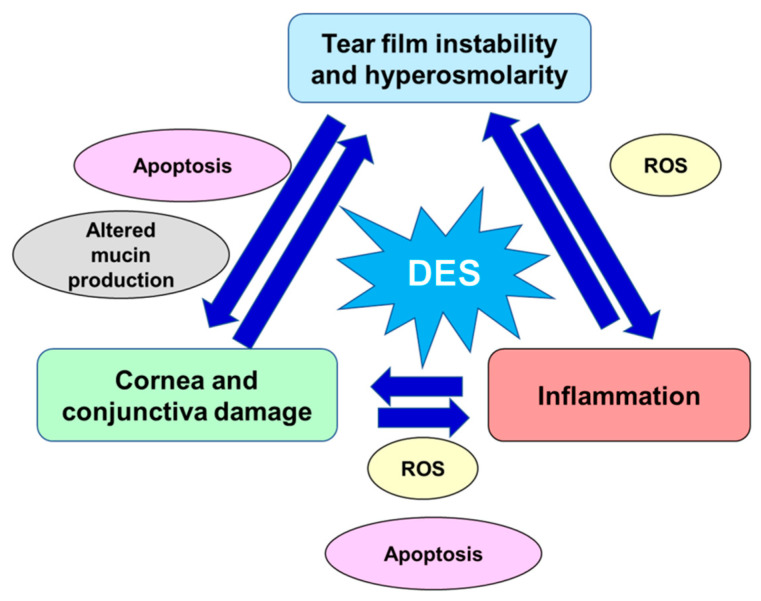
Key pathogenic factors contributing to the pathological vicious circle of dry eye disease. DED: dry eye disease; ROS: reactive oxygen species.

Furthermore, the activity of caspases 8, 9, 3, and 7 was enhanced in hyperosmotic conditions inducing apoptotic processes at the corneal/conjunctival level [33,34,35]. In the conjunctival mucosa, a goblet cell loss, directly related to chronic inflammation and apoptosis processes and induced by tear film hyperosmolarity and epithelial damage, might result in further and important tear film instability [24,25,36]. Moreover, the ocular surface altered by the apoptotic process together with hyperosmolar environment, reduces the stability of the tear film [37] and excites the corneal nerve fibers, which may lead to blinking, stimulation of lacrimal gland, and other DED symptoms [38]. Damage to the conjunctival epithelium significantly impaired mucins production, glycoproteins responsible for binding water and keeping the ocular surface hydrated, resulting in the inability to create a stable and normosmolar tear film [39,40]. In Figure 1, are schematized the key pathogenic factors contributing to the pathological downward spiral of DED. The ocular surface stresses that can initiate this cycle are variable and innumerous.

Among the systemic drugs that may cause DED, many are best-selling ones, which may decrease tear production, alter nerve function, induce inflammatory effects on secretory glands without reaching the ocular surface or may have direct irritation effects through secretion into the tears. In fact, the 62% of DED cases in the elderly are secondary to medications, including nonsteroidal anti-inflammatory drugs, diuretics, vasodilators, analgesics/antipyretics, antiulcer agents, sulfonylureas, cardiac glycosides, anxiolytics/benzodiazepines, anti-infectives, antidepressants/antipsychotics, hypotensive agents, and antihistamines [12,41,42].

DED patients can have the perception of a foreign body in the eye, sore eyes, and reduced visual acuity, compromising the ability of the individual to concentrate, particularly for the ones working at a computer monitor [43,44]. Even if the symptoms are not dangerous, they should not be underestimated, as most of the patients stated an important reduction in quality of life.

### 1.2. Therapy of Dry Eye Disease

Some of the possible therapeutic strategies for improving symptoms of DED are reducing tear evaporation and absorption, increasing or supplementing tear production, and reducing ocular inflammation and oxidative stress [45]. The first-line treatments for this pathology are tear supplementation, environmental strategies, heat application to soften secretions in obstructed tear glands, correction of eventual eyelid anatomical defects, or discontinuation of DED-inducing drugs [24,46,47]. To date, many randomized clinical trials evaluating DED treatments have been performed searching for transparent diagnostic criteria and standardized outcomes. Laser-assisted in situ keratomileusis (LASIK) is a refractive procedure, which is associated with a 20% of chronic dry eye [48]. This relationship between LASIK and DED makes such patients a valuable population for clinical trials. After a LASIK intervention, the main therapy is the use of artificial tears, which are normally composed of cellulose to maintain viscosity, a spreading agent like polyethylene glycol to prevent evaporation and preservative agents, like benzalkonium chloride, to prevent bacterial and fungus contamination [49]. The artificial tears can contain also aqueous supplementing molecules like carboxymethycellulose and sodium hyaluronate or polar phospholipids like dimyristoylphosphatidylglycerol [50]. The application of artificial tears is usually not curative and requires chronic applications, unless a specific etiologic agent is temporary or can be eliminated. Some behavioral attentions, like frequently blinking and avoiding air conditioning, contribute to alleviating the patients’ DED discomfort. Conversely, humidifiers in close rooms can sometimes improve the symptom [51]. As reported in the TFOS DEWS II Management and Therapy Report, DED treatment progresses in a stepwise approach, starting with patient education, environmental modification, lid hygiene, and artificial tears application [50]. As previously reported, artificial tears of various types are the mainstay of DED management. Due to the staged management of DED, if the initial steps are inadequate, it is possible to consider other devices (e.g., pulsed light therapy, lifitegrast, topical secretagogues, etc.) which may be used in conjunction with prescription medications [50]. In order to improve tear production, secretagogues agents can be used, like rebamipide, a mucin inducer, or diquafosol tetrasodium [52,53]. Interestingly, also some antibiotics can be used as DED therapy. In particular, tetracycline showed important effects on MMPs, lipases, and pro-inflammatory cytokines [54]; whereas, azithromycin showed empirical secretory effects on Meibomian glands [55]. Other relevant agents against DED are integrin lymphocyte function-associated antigen-1 (LFA-1) antagonist drugs (such as lifitegrast). Lifitegrast acts as a competitive antagonist which blocks the binding between LFA-1 and intracellular adhesion molecule-1 and thereby inhibits T cell migration and inflammatory cascade activation [23,56,57]. In DED non-responding states, the application of corticosteroids or cyclosporin A may be considered. However, corticosteroids should be used only for a short time, as the elevation of intraocular pressure and formation of cataract occur during long-term use [58,59] and cyclosporin A, cyclic polypeptide that prevents T cell maturation, seems low effective and it may induce severe side effects, such as itching, eye redness, and blurred vision [60,61,62]. Furthermore, due to its’ hydrophobic structure, cyclosporin A presents poor biopharmaceutical properties, such as low solubility and permeability, so resulting in difficulties in the formulation and efficient drug delivery [63]. Another possible step-approach against DED is intense pulsed light that seems an effective and safe DED treatment option; in fact, it improves tear film quality and reduces DED symptoms and related discomfort [64].

A new therapy should be quested targeting not only inflammatory biochemical mechanisms, but also oxidative stress, ocular surface injury, and excessive irritation of corneal afferent neurons. In this regard, as described in the following paragraphs, polyphenols represent natural and potent agents potentially effective against DED. In particular, in the present review, was conducted a deep literature search focusing on the main polyphenols tested against DED and analyzing related in vitro, in vivo studies, and clinical trials (to date just one). In the following paragraphs, the tested polyphenols are introduced and outlined, their currently known effects and mechanisms of action against DED, underlining their therapeutic potential.

### 1.3. Polyphenols

**Figure 2:**
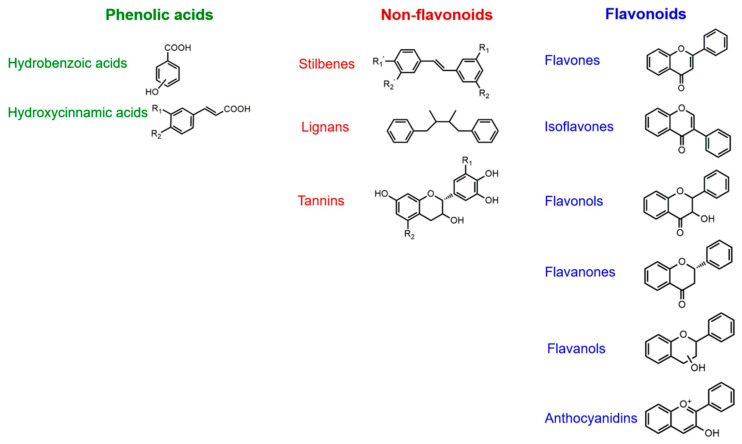
Classification of polyphenols in three classes based on their structure: phenolic acids, non-flavonoids, and flavonoids.

Polyphenols are natural compounds that originate exclusively as plant secondary metabolites [65,66]. They are characterized by one or more phenol rings on which basis they are classified (Figure 2). Due to their structure, they are effective antioxidant molecules able to decrease the production of free radicals through Fenton reaction [67,68,69]. Moreover, polyphenols can directly interact with various enzymes, resulting in anti-inflammatory, anti-microbial, anti-viral, anti-aging, anticancer properties and they also show neuroprotective effects [70,71,72,73,74,75].

Polyphenols showed therapeutic effects in various fields and the most studied molecules are epigallocatechin gallate (EGCG), resveratrol, quercetin, betaine, pterostilbene, and curcumin [76,77,78,79]. Hence, evident therapeutic effects have been confirmed, despite the bioavailability of polyphenols which is, in general, low [80,81,82]. Notably, the oral administration of polyphenols is safe [83,84]. To date, topical application of polyphenols showed an important potential as treatment of DED [77,85,86,87].

## 2. Polyphenols’ Effects in DED

Principally, there are two methods for in vitro DED induction: either treatment of corneal cells with interleukin-1β (IL-1β) or with tumor necrosis factor-α (TNF-α) or increasing the osmolarity of culture medium using a hyperosmolar medium. The remarkable advantage of in vivo studies, compared to in vitro studies, is the possibility to evaluate not only the biomarkers of DED pathological mechanisms, but also the same parameters which are examined at the ophthalmological visit, such as tear fluid production or fluorescein staining. This last procedure consists of the application of fluorescein solution in the patient’s eye, permitting the observation of corneal epithelial defects with the use of a slit lamp [88]. The methods of DED induction to an animal include several topical treatments or the chambers with the desiccating stress conditions. To date, just one clinical trial was performed in DED patients to test the effects of the polyphenol EGCG, but it represents an important starting point to reinforce polyphenols’ role in the treatment of DED. However, more clinical trials are needed in the next future, assessing the best combinations and dosages for the patient.

**Figure 3:**
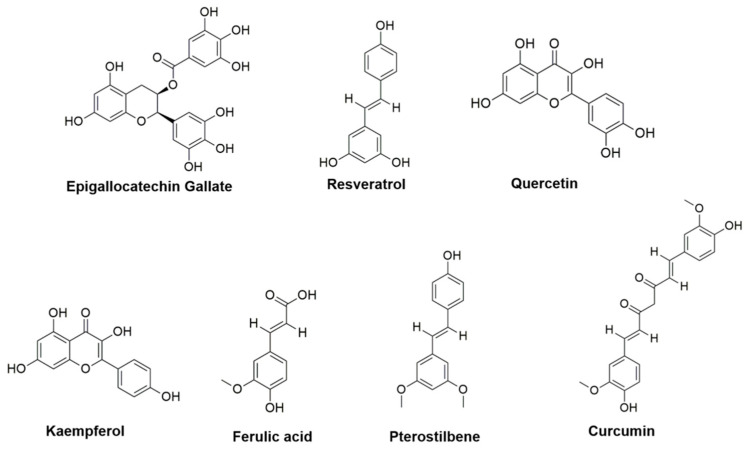
Polyphenols actually tested against dry eye disease: epigallocatechin gallate, resveratrol, quercetin, kaempferol, ferulic acid, pterostilbene, and curcumin.

In the following paragraphs, the results and observations from in vitro, in vivo, and human studies about the effects of topically applied polyphenolic compounds against DED are reviewed, underlining their mechanisms of action and beneficial effects against the main DED injuries and related symptoms. In Figure 3, are schematized the polyphenols described in the following paragraphs.

### 2.1. Epigallocatechin Gallate

EGCG is the most abundant catechin present in green tea and it is believed to be responsible for most of the biological beneficial effects of tea [89]. EGCG is an active catechin showing antioxidant, anti-inflammatory, epigenetic, low-density lipoprotein cholesterol lowering properties, and also anticancer effects [90,91,92,93,94,95]. Shim et al. [96] described how this catechin could be solubilized, remedying its poor aqueous solubility.

#### 2.1.1. In Vitro Studies

For evaluating EGCG potential as DED treatment, human corneal epithelial cells (HCECs) were used and dose-dependent inhibition of the inflammatory metabolic pathway was confirmed. Specifically, decrease in pro-inflammatory cytokines was observed after 30 µM of EGCG treatment, even if inhibition of the kinases and transcription factors leading to pro-inflammatory cytokine release (phosphorylation of MAPKs p38 unit and JNK and inhibition of NF-kB and AP-1 activities) already occurred at 3 µM of EGCG. Furthermore, the index of cell viability confirmed no harmful effect of 30 µM EGCG concentration. The EGCG treatment of HCEC lasted for 18 hours [97], less than Luo and Lai [98] who treated cells for 3 days with 0.1% EGCG loaded gelatin polymer and showed normal cell morphology, proliferative capacity, and viability in the control-gelatin group and gelatin-EGCG group. After DED induction followed with EGCG treatment, the authors also observed down-regulation of pro-inflammatory cytokines and chemokines and significant inhibition of ROS. EGCG also inhibited ROS in human corneal limbal epithelium with half-maximal effective concentration (EC50) equaled to 3.41 µM after 60 min of treatment [99].

#### 2.1.2. In Vivo Studies

A mice DED model was treated with 0.1% EGCG after 48 hours of DED induction, observing a significant decrease in fluorescein corneal staining (analyses normally used in clinical practice for detection of foreign bodies in the eye or abrasions of the cornea) and also of pro-inflammatory cytokines and vascular endothelial growth factor. The level of TNF-α was not influenced, but apoptosis of corneal epithelium has been inhibited [100]. In the study of Tseng et al. [101], 0.001% EGCG enriched artificial tears tested on the rabbit DED model were not sufficient to significantly restore a normal tear fluid production after a 3-week application, but the combination with hyaluronic acid reached values comparable to healthy controls. Notably, a significant decrease in inflammatory activity was also described in the group with mixed treatment in comparison with the one treated with hyaluronic acid alone. Recently, Luo et al. [102] developed a long-acting and functional ophthalmic formulation for the treatment of DED. In detail, the experimental rabbit model of DED was treated with one-time topical instillation of a biodegradable, mucoadhesive, and thermo-responsive hydrogel that provides a sustained release of EGCG and increases the “drug” bioavailability to therapeutic levels for a period over 14 days. Luo et al. [102] observed that this specific formulation in the conjunctival sac could effectively repair corneal epithelial defects through the attenuation of cellular inflammation, oxidative stress, and apoptosis. Interestingly, these findings on a long-acting pharmaceutical formulation underlined the possible management of this complex ocular disease via a simple topical administration route. Luo and Lai [98] studied EGCG loaded gelatin-g-poly(N-isopropylacrylamide). In this form, EGCG was released continuously and, after 6 hours, significantly improved fluorescein and Bengal rose staining scores (a clinically commonly used method to stain damaged conjunctival and corneal cells and thereby identify damages at the ocular surface level). In the group with EGCG in gelatin polymer, the aqueous part of tear film together with epithelial thickness was significantly restored in comparison with the application of either gelatin polymer or EGCG alone. Furthermore, a significant loss of goblet cells, which are responsible for gel-forming mucins secretion in the conjunctiva, was detected in all groups treated with the gelatin carrier alone, EGCG, or EGCG in gelatin polymer. Similarly, Huang et al. [103] studied the gelatin-EGCG nanoparticles coated with hyaluronic acid in phosphate buffer (GEH). The optimal EGCG concentration was defined as 20 µg/mL (0.002%). The formula enabled longer retention on the ocular surface and showed a higher cellular uptake. The rabbit model of DED was treated with GEH twice daily for 3 weeks, showing that the tear secretion was not significantly recovered, but on the other hand, fluorescein staining exhibited lower scores and a lower amount of corneal apoptotic cells were determined. Moreover, inflammatory cytokines significantly decreased after EGCG and GEH treatment.

To further improve drug accessibility to the ocular surface, Li et al. [95] prepared polycatechin-capped gold nanoparticles transporting amfenac, which is part of the family of non-steroidal anti-inflammatory drugs. Catechin and catechin nanoparticles without amfenac topically administrated to rabbit DED models showed significant improvement in corneal thickness, corneal fluorescein staining and Bengal rose staining. Moreover, a reduction in corneal apoptosis was detected together with an increased amount of goblet cells suggesting a therapeutic role of catechin.

#### 2.1.3. Clinical Trial

Nejabat et al. [104] evaluated green tea extract rich in EGCG as topical DED treatment in a double-blind randomized controlled clinical trial with 60 patients involved. Patients followed the treatment for one month. A significant subjective symptom alleviation was recorded in the group with the green tea extract drops. Schirmer test, corneal and conjunctival staining did not show significant differences between the groups, but considerable improvement in tear break-up time (TBUT) test and meibum quality were detected suggesting a progress in tear film stability. Interestingly, EGCG was well tolerated, representing a safe topical treatment against DED.

### 2.2. Resveratrol

Resveratrol is a natural phytoalexin polyphenol that is found in more than 70 species of plants, herbs, fruits, or vegetables, like mulberries, peanuts, grape, and berry skins [105]. It is well-absorbed and quickly metabolized in the body [106]. Resveratrol is generally known to be beneficial for human health, in fact, several biological effects have been reported, including anticancer activity, anti-inflammatory, antioxidant, cardio- and neuro-protective effects [106,107,108]. It seems so beneficial for several pathological processes like cardiovascular and neurodegenerative diseases, tumors, pain, tissue injury, and inflammatory-related diseases [109].

#### 2.2.1. In Vitro Studies

Resveratrol is another bioactive polyphenol tested against DED. Using a solution containing 50 µM of resveratrol in the DED cell model for 24 hours, Abengózar-Vela and colleagues [85] observed a significant reduction in the levels of pro-inflammatory cytokines IL-6, -8, and interferon gamma-induced protein-10. Recently, Shetty et al. [110] investigated the role of resveratrol on vitamin D levels and Notch signaling under hyperosmolar conditions. HCECs in hyperosmolar conditions had increased reactive oxygen species levels and decreased vitamin D levels that got restored, most likely through activation of Notch signaling, in the presence of resveratrol. However, an understanding of the molecular basis of corneal epithelial phenotype and Notch signaling modulation to restore corneal surface integrity and health is needed.

#### 2.2.2. In Vivo Study

Abengózar-Vela et al. [111] applied a 0.1% resveratrol solution 3 times a day, starting one day before DED induction, in a mice model. Such treatment improved the fluorescein staining score; however, it did not enhance tears production. On day 6, pro-inflammatory cytokines in the tear fluid were measured and the pro-inflammatory IL-1α showed a significant decrease together with a notably reversed CD4+ T cells infiltration in the resveratrol treated group.

### 2.3. Quercetin

Quercetin is a flavonoid abundantly present in a wide variety of fruits and vegetables as well as in tea and red wine [112,113,114,115]. It is also found in medicinal botanicals, including Ginkgo biloba, Hypericum Perforatum, and Sambucus Canadensis [116]. In a typical Western diet, the daily intake of quercetin is estimated to be in the range of 0 and 30 mg [112]. Quercetin shows unique biological properties at the basis of potential benefits to overall health and disease resistance, in fact, it exhibits anticarcinogenic, anti-inflammatory, antiviral, anti-hypertensive, and antioxidant effects as well as it may inhibit lipid peroxidation and platelet aggregation and it can stimulate mitochondrial biogenesis [114,116,117,118].

#### 2.3.1. In Vitro Study

In the last years, the implementation of quercetin as a therapeutic application to the ocular surface has been gaining the attention of the scientific community in the study of DED, keratoconus, and injured cornea [119,120]. Abengózar-Vela et al. [85] evaluated the effects of quercetin or quercetin plus resveratrol treatments obtaining a significant anti-inflammatory and antioxidant effects. Furthermore, quercetin showed a relevant potential in the inhibition of ROS in comparison with other polyphenols, showing an EC50 of 2.98 µM [99].

#### 2.3.2. In Vivo Studies

Oh et al. [120] observed in DED mice, treated with 0.5% quercetin 4 times per day, a significant tear volume restoration to the basal level after 3 days, that interestingly reached the plateau at the 7th day of therapy. Quercetin treatment significantly improved the corneal surface irregularities from the 3rd day of application. Furthermore, it significantly decreased the detachment of corneal cells and increased the number of conjunctival goblet cells. The quercetin protective effect was also shown against TNF-α, MMP-2, MMP-9, intercellular adhesion molecule-1, and vascular cell adhesion molecule-1, which were significantly decreased in the group treated with quercetin. On the other hand, Abengózar-Vela et al. [111] used only 0.01% quercetin solution applied 3 times per day starting one day before DED induction to mice and, after 9 days, the positive effects were detected with a significant improvement of the corneal surface, but without enhancement in tear volume.

### 2.4. Kaempferol and Ferulic Acid

Kaempferol is a ubiquitous polyphenol, present in fruits and vegetables. The richest plant sources of kaempferol are green leafy vegetables, such as spinach, kale, and herbs including dill, chives, and tarragon [121]. This polyphenol shows antioxidant, anti-inflammatory, cardioprotective, and anticancer properties [121,122,123]. However, still further clinical trials must be carried out to confirm its beneficial effects during clinical application at the aim to manage inflammatory-related diseases [124]. To date, few human studies investigated kaempferol bioavailability and reported that kaempferol glucoside and rutinoside in tea were the most bioavailable forms of this polyphenol.

Ferulic acid is a common polyphenolic molecule most abundantly present in vegetables, like artichokes, eggplants, and in maize bran. Furthermore, it is an effective component of Chinese medicinal herbs such as Angelica Sinensis, Cimicifuga Heracleifolia, and Lignsticum Chuangxiong [125]. Ferulic acid is a polyphenolic compound that possesses excellent antioxidant properties [99,126,127]. However, it is important to underline that this latter is not the only interesting property of this polyphenol, due to various studies, exhibited ferulic acid cytoprotective effects [125,128,129]. To date, ferulic acid shows a poor bioavailability that, together with concerns derived from its pharmacokinetics, may be limit the potential use of ferulic acid in clinical application.

#### In Vivo Study

Chen et al. [122] studied the combination of two polyphenolic compounds: kaempferol and ferulic acid. The optimal combination has been experimentally set as 100 µM of ferulic acid plus 1 µM of kaempferol. This polyphenolic combination was used in the rabbit DED model, which was treated 3 times per day for 3 weeks. Significant tear fluid restoration was detected together with fluorescein staining improvement as also corneal thickness recovery [122].

To date, there are no specific in vitro studies nor clinical trials about ferulic acid and/or kaempferol against DED.

### 2.5. Pterostilbene

Pterostilbene is a naturally-derived stilbenoid that structurally resembles resveratrol. It was initially isolated from sandalwood, but is also present in fruits, including grapes and blueberries [130]. The pterostilbene wide range of bioactive properties comprehends antioxidant, anti-inflammatory, anticancer, and cardioprotective effects [131,132,133,134], which altogether may contribute to the prevention of various chronic human diseases. Moreover, it seems to improve cognitive performances [135].

#### In Vitro Studies

In the HCEC DED model, 5 µM of pterostilbene concentration significantly decreased the expression of IL-6, IL-1β, and TNF-α on both protein and mRNA level. Furthermore, it downregulated some MMPs together with markers of lipid peroxidation and mitochondrial damage. Pterostilbene protected HCECs against ROS overproduction too, and significantly increased the endogenous antioxidant enzymes SOD1 and peroxiredoxin 4 [136]. Recently, Hu et al. [137] designed and synthesized a pterostilbene-peptide amphiphile which could spontaneously self-assemble into nanomedicine in an aqueous solution to improve water solubility. The main objective of this study was to develop a pterostilbene prodrug nanomedicine with improved solubility and reduced toxicity for the potential management of DED. Based on this pilot in vitro study, the synthesized pterostilbene nanomedicine showed both anti-inflammatory effects and potent ROS scavenging activity, which might be promising in ocular drug development.

To date, there are no in vivo studies or clinical trials about pterostilbene against DED.

### 2.6. Curcumin

Curcumin is one of the most studied polyphenols in the turmeric spice. Curcumin exhibits a broad range of biological and pharmacological activities, including anti-inflammatory, antioxidant, anti-tumor, chemosensitizing, epatoprotective, lipid-modifying, and neuroprotective effects [138,139,140]. Curcumin and its derivatives may potentially ameliorate the hallmarks of neurodegenerative diseases [141], but further clinical studies are needed. Curcumin presents poor bioavailability and low gastrointestinal absorption which is mainly attributed to water insolubility, rapid metabolism, and excretion [142,143].

#### 2.6.1. In Vitro Study

Chen et al. [144] observed in hyperosmotic-stressed HCECs, that 5 µM curcumin induced significant anti-inflammatory effects both on mRNA and the protein level. Additionally, downregulation of MAPK and JNK, which regulates inflammatory cytokines production, and NF-kB transcription factor were observed.

#### 2.6.2. In Vivo Study

Muz et al. [145] evaluated, in a DED animal model, the potential protective effects of an oral formulation composed by lutein/zeaxanthin, curcumin, and vitamin D3 (LCD). The LCD was administered, at different doses, to benzalkonium chloride-induced DED animal model for 4 weeks. LCD formulation significantly limited the inflammatory cascade and oxidative stress, together with ameliorating tear secretion and recovering neurotrophic factors and glycocalyx expression.

To date, there are no clinical studies about treatment with curcumin against DED.

## 3. Conclusions

A lot of focus has been put on the pathophysiology and possible therapies of DED, but still the current knowledge seems not to be enough to exhaustively help the clinical practice to efficiently manage its symptoms. Polyphenols are natural compounds with high efficacy to suppress oxidative stress and inflammation. They showed high potential in the treatment of neoplastic diseases and did not show important side effects. As their bioavailability is low, application avoiding the gastrointestinal tract is recommended [146,147].

Based on our deep review of the literature, topically applied polyphenols present positive and promising outcomes also against DED (Table 1). Currently, DED is treated with artificial tears, not targeting its pathophysiological mechanisms, or with anti-inflammatory agents that showed important side effects. Polyphenols, targeting the core mechanisms of DED, could be a novel and effective topical treatment against DED. Another interesting and not negligible way to contrast DED symptoms could be by dietetic regimens. Different micronutrients and nutraceutical products showed an important role against ocular surface disease [30] and caloric restriction has been shown to recover age-related functional declines in various organs, including the eye [148]. Although the oral route did not show an optimal absorption, a diet focused on the uptake of polyphenols could show benefits on patients with DED. However, randomized clinical trials are fundamental to affirm definitely the efficacy and safety of a specific dietetic regimen.

## Figures and Tables

**Table 1:** Evidence of activity of polyphenols against dry eye diseases.

Polyphenoles	In Vitro Studies		In Vivo Studies					Clinical Trial
	Anti-Inflammatory Effect	Antioxidant Effect	Anti-Inflammatory Effect	Antioxidant Effect	Anti-Apoptotic Effect	Improvement of Ocular Surface Damage	Tear Production	Tear Film Stability
Curcumin	[144]		[145]					
Epigallocatechin Gallate	[97,98]		[95,98,100,101,102,103]	[95,98,102]	[95,100,101,102,103]	[95,98,100,101,102,103]	[95,101,103]	[104]
Ferulic Acid and Kaempferol			[122]					
Pterolstilbene	[136]							
Quercetin	[85]		[111,120]			[111,120]	[120]	
Resveratrol	[85,110]		[111]			[111]		

## References

[1] Shimazaki J. (2018). Definition and diagnostic criteria of dry eye disease: Historical overview and future directions. Investig. Ophthalmol. Vis. Sci..

[2] Wolffsohn J.S., Arita R., Chalmers R., Djalilian A., Dogru M., Dumbleton K., Gupta P.K., Karpecki P., Lazreg S., Pult H. (2017). TFOS DEWS II diagnostic methodology report. Ocul. Surf..

[3] Bron A.J., de Paiva C.S., Chauhan S.K. (2017). TFOS DEWS II pathophysiology report. Ocul. Surf..

[4] Farrand K.F., Fridman M., Stillman I.Ö., Schaumberg D.A. (2017). Prevalence of diagnosed dry eye disease in the United States among adults aged 18 years and older. Am. J. Ophthalmol..

[5] Titiyal J.S., Falera R.C., Kaur M., Sharma V., Sharma N. (2018). Prevalence and risk factors of dry eye disease in North India: Ocular surface disease index-based cross-sectional hospital study. Indian J. Ophthalmol..

[6] Clegg J.P., Guest J.F., Lehman A., Smith A.F. (2006). The annual cost of dry eye syndrome in France, Germany, Italy, Spain, Sweden and the United Kingdom among patients managed by ophthalmologists. Ophthalmic Epidemiol..

[7] Kim J.S., Wang M.T.M., Craig J.P. (2019). Exploring the Asian ethnic predisposition to dry eye disease in a pediatric population. Ocul. Surf..

[8] De Paiva C.S. (2017). Effects of aging in dry eye. Int. Ophthalmol. Clin..

[9] Craig J.P., Wang M.T.M., Ambler A., Cheyne K., Wilson G.A. (2020). Characterising the ocular surface and tear film in a population-based birth cohort of 45-year old New Zealand men and women. Ocul. Surf..

[10] Peck T., Olsakovsky L., Aggarwal S. (2017). Dry eye syndrome in menopause and perimenopausal age group. J. Midlife Health.

[11] Wang H., Wang P.-B., Chen T., Zou J., Li Y.-J., Ran X.-F., Lin Q.X. (2017). Analysis of clinical characteristics of immune-related dry eye. J. Ophthalmol..

[12] Fraunfelder F.T., Sciubba J.J., Mathers W.D. (2012). The role of medications in causing dry eye. J. Ophthalmol..

[13] Kitazawa M., Sakamoto C., Yoshimura M., Kawashima M., Inoue S., Mimura M., Subota K., Negishi K., Kishimoto T. (2018). The relationship of dry eye disease with depression and anxiety: A naturalistic observational study. Transl. Vis. Sci. Technol..

[14] Kojima T. (2018). Contact lens-associated dry eye disease: Recent advances worldwide and in Japan. Investig. Ophthalmol. Vis. Sci..

[15] Demirci G., Karaman E., Ozsutcu M., Eliacik M., Olmuscelik O., Aydin R., Kocabora M.S. (2018). Dry eye assessment in patients with vitamin D deficiency. Eye Contact Lens.

[16] Lee M.H., Sarossy M.G., Zamir E. (2015). Vitamin A deficiency presenting with ‘Itchy Eyes’. Case Rep. Ophthalmol..

[17] Tandon R., Vashist P., Gupta N., Gupta V., Sahay P., Deka D., Singh S., Vishwanath K., Murthy G.V.S. (2020). Association of dry eye disease and sun exposure in geographically diverse adult (≥40 years) populations of India: The SEED (sun exposure, environment and dry eye disease) study—Second report of the ICMR-EYE SEE study group. Ocul. Surf..

[18] Paulsen A.J., Cruickshanks K.J., Fischer M.E. (2014). Dry eye in the beaver dam offspring study: Prevalence, risk factors, and health-related quality of life. Am. J. Ophthalmol..

[19] Sullivan D.A., Rocha E.M., Aragona P., Clayton J.A., Ding J., Golebiowski B., Hampel U., McDermott A.M., Schaumberg D.A., Srinivasan S. (2017). TFOS DEWS II Sex, gender, and hormones report. Ocul. Surf..

[20] Li W., Lin M.C. (2019). Sex disparity in how pain sensitivity influences dry eye symptoms. Cornea.

[21] Calonge M., Pinto-Fraga J., González-García M.J. (2017). Effects of the external environment on dry eye disease. Int. Ophthalmol. Clin..

[22] Jamerson E.C., Elhusseiny A.M., ElSheikh R.H., Eleiwa T.K., El Sayed Y.M. (2020). Role of matrix metalloproteinase 9 in ocular surface disorders. Eye Contact Lens.

[23] Gupta P.K., Asbell M.P., Sheppard M.J. (2020). Current and future pharmacological therapies for the management of dry eye. Eye Contact Lens.

[24] Aragona P., Giannaccare G., Mencucci R., Rubino P., Cantera E., Rolando M. (2020). Modern approach to the treatment of dry eye, a complex multifactorial disease: A P.I.C.A.S.S.O. board review. Br. J. Ophthalmol..

[25] Aragona P., Rolando M. (2013). Towards a dynamic customised therapy for ocular surface dysfunctions. Br. J. Ophthalmol..

[26] Hong S.C., Ha J.H., Lee J.K., Jung S.H., Kim J.C. (2020). In vivo anti-inflammation potential of aster koraiensis extract for dry eye syndrome by the protection of ocular surface. Nutrients.

[27] Wei Y., Asbell P.A. (2014). The core mechanism of dry eye disease is inflammation. Eye Contact Lens.

[28] Deng R., Hua X., Li J., Chi W., Zhang Z., Lu F., Zhang L., Pflugfelder S.C., Li D.Q. (2015). Oxidative stress markers induced by hyperosmolarity in primary human corneal epithelial cells. PloS ONE.

[29] Park B., Jo K., Lee T.G., Hyun S.W., Kim J.S., Kim C.S. (2019). Polydatin Inhibits NLRP3 inflammasome in dry eye disease by attenuating oxidative stress and inhibiting the NF-κB pathway. Nutrients.

[30] Pellegrini M., Senni C., Bernabei F., Cicero A.F.G., Vagge A., Maestri A., Scorcia V., Giannaccare G. (2020). The role of nutrition and nutritional supplements in ocular surface diseases. Nutrients.

[31] Saccà S.C., Cutolo C.A., Ferrari D., Corazza P., Traverso C.E. (2018). The eye, oxidative damage and polyunsaturated fatty acids. Nutrients.

[32] Žiniauskaitė A., Ragauskas S., Ghosh A.K., Thapa R., Roessler A.E., Koulen P., Kalesnykas G., Hakkarainen J.J., Kaja S. (2019). Manganese(III) tetrakis(1-methyl-4-pyridyl) porphyrin, a superoxide dismutase mimetic, reduces disease severity in in vitro and in vivo models for dry-eye disease. Ocul. Surf..

[33] Khandekar N., Willcox M.D.P., Shih S., Simmons P., Vehige J., Garrett Q. (2013). Decrease in hyperosmotic stress–induced corneal epithelial cell apoptosis by L-carnitine. Mol. Vis..

[34] Rybickova I., Vesela V., Fales I., Skalicka P., Jirsova K. (2016). Apoptosis of conjunctival epithelial cells before and after the application of autologous serum eye drops in severe dry eye disease. Biomed. Pap. Med. Fac. Univ. Palacky. Olomouc. Czech. Repub..

[35] Yamaguchi T. (2018). Inflammatory response in dry eye. Investig. Ophthalmol. Vis. Sci..

[36] Zhang X., Chen W., De Paiva C.S., Corrales R.M., Volpe E.A., McClellan A.J., Farley W.J., Li D.Q., Pflugfelder S.C. (2011). Interferon-γ exacerbates dry eye-induced apoptosis in conjunctiva through dual apoptotic pathways. Investig. Ophthalmol. Vis. Sci..

[37] Pflugfelder S.C., de Paiva C.S. (2017). The pathophysiology of dry eye disease: What we know and future directions for research. Ophthalmology.

[38] Labetoulle M., Baudouin C., Calonge M., Merayo-Lloves J., Boboridis K.G., Akova Y.A., Aragona P., Geerling G., Messmer E.M., Benítez-Del-Castillo J. (2019). Role of corneal nerves in ocular surface homeostasis and disease. Acta Ophthalmol..

[39] Ablamowicz A.F., Nichols J.J. (2016). Ocular surface membrane-associated mucins. Ocul. Surf..

[40] Baudouin C., Rolando M., Del Castillo J.M.B., Messmer E.M., Figueiredo F.C., Irkec M., Van Setten G., Labetoulle M. (2019). Reconsidering the central role of mucins in dry eye and ocular surface diseases. Prog. Retin. Eye Res..

[41] Gomes J.A.P., Azar D.T., Baudouin C., Efron N., Hirayama M., Horwath-Winter J., Kim T., Mehta J.S., Messmer E.M., Pepose J.S. (2017). TFOS DEWS II iatrogenic report. Ocul. Surf..

[42] Schein O.D., Hochberg M.C., Muñoz B., Tielsch J.M., Bandeen-Roche K., Provost T., Anhalt G.J., West S. (1999). Dry eye and dry mouth in the elderly: A population-based assessment. Arch. Int. Med..

[43] Clayton J.A. (2018). Dry eye. N. Engl. J. Med..

[44] Okumura Y., Inomata T., Iwata N., Sung J., Fujimoto K., Fujio K., Midorikawa-Inomata A., Miura M., Akasaki Y., Murakami A. (2020). A Review of dry eye questionnaires: Measuring patient-reported outcomes and health-related quality of life. Diagnostics.

[45] Gomes J.A.P., Santo R.M. (2019). The impact of dry eye disease treatment on patient satisfaction and quality of life: A review. Ocul. Surf..

[46] lves M., Fonseca E.C., Alves M.F., Malki L.T., Arruda G.V., Reinach P.S., Rocha E.M. (2013). Dry eye disease treatment: A systematic review of published trials and a critical appraisal of therapeutic strategies. Ocul. Surf..

[47] Marshall L.L., Roach J.M. (2016). Treatment of dry eye disease. Consult. Pharm..

[48] Shoja M.R., Besharati M.R. (2007). Dry eye after LASIK for myopia: Incidence and risk factors. Eur. J. Ophthalmol..

[49] Pucker A.D., Ng S.M., Nichols J.J. (2016). Over the counter (OTC) artificial tear drops for dry eye syndrome. Cochrane Database Syst. Rev..

[50] Jones L., Downie L.E., Korb D. (2017). TFOS DEWS II management and therapy report. Ocul. Surf..

[51] Wang M.T.M., Chan E., Ea L., Kam C., Lu Y., Misra S.L., Craig J.P. (2017). Randomized trial of desktop humidifier for dry eye relief in computer users. Optom. Vis. Sci..

[52] Dogru M., Nakamura M., Shimazaki J., Tsubota K. (2013). Changing trends in the treatment of dry-eye disease. Expert Opin. Investig. Drugs..

[53] Itoh S., Itoh K., Shinohara H. (2014). Regulation of human corneal epithelial mucins by rebamipide. Curr. Eye Res..

[54] De Paiva C.S., Corrales R.M., Villarreal A.L., Farley W.J., Li D.Q., Stern M.E., Pflugfelder S.C. (2006). Corticosteroid and doxycycline suppress MMP-9 and inflammatory cytokine expression, MAPK activation in the corneal epithelium in experimental dry eye. Exp. Eye Res..

[55] Liu Y., Kam W.R., Ding J., Sullivan D.A. (2015). Can tetracycline antibiotics duplicate the ability of azithromycin to stimulate human meibomian gland epithelial cell differentiation?. Cornea.

[56] Abidi A., Shukla P., Ahmad A. (2016). Lifitegrast: A novel drug for treatment of dry eye disease. J. Pharmacol. Pharmacother..

[57] Sheppard J.D., Torkildsen G.L., Lonsdale J.D., D’Ambrosio F.A., McLaurin E.B., Eiferman R.A., Kennedy K.S., Semba C.P., OPUS-1 Study Group (2014). Lifitegrast ophthalmic solution 5.0% for treatment of dry eye disease: Results of the OPUS-1 phase 3 study. Ophthalmology.

[58] Cutolo C.A., Barabino S., Bonzano C., Traverso C.E. (2019). The use of topical corticosteroids for treatment of dry eye syndrome. Ocul. Immunol. Inflamm..

[59] Rice M.L., Wong B., Horn P.S., Yang M.B. (2018). Cataract development associated with long-term glucocorticoid therapy in Duchenne muscular dystrophy patients. J. AAPOS.

[60] Kim Y.J., Ryu J.S., Park S.Y., Lee H.J., Ko J.H., Kim M.K., Wee W.R., Oh J.Y. (2016). Comparison of topical application of TSG-6, cyclosporine, and prednisolone for treating dry eye. Cornea.

[61] Rao S.N. (2011). Reversibility of dry eye deceleration after topical cyclosporine 0.05% withdrawal. J. Ocul. Pharmacol. Ther..

[62] Schultz C. (2014). Safety and efficacy of cyclosporine in the treatment of chronic dry eye. Ophthalmol. Eye Dis..

[63] Yavuz B., Bozdağ Pehlivan S., Kaffashi A., Çalamak S., Ulubayram K., Palaska E., Çakmak H.B., Ünlü N. (2016). In vivo tissue distribution and efficacy studies for cyclosporin A loaded nano-decorated subconjunctival implants. Drug Deliv..

[64] Vergés C., Salgado-Borges J., Ribot F.M. (2020). Prospective evaluation of a new intense pulsed light, thermaeye plus, in the treatment of dry eye disease due to meibomian gland dysfunction. J. Optom..

[65] Delgado S.R., Arbelaez A.F.A., Rojano B. (2019). Antioxidant capacity, bioactive compounds in coffee pulp and implementation in the production of infusions. Acta Sci. Pol. Technol. Aliment..

[66] Singla R.K., Dubey A.K., Garg A., Sharma R.K., Fiorino M., Ameen S.M., Haddad M.A., Al-Hiary M. (2019). Natural polyphenols: Chemical classification, definition of classes, subcategories, and structures. J. AOAC Int..

[67] Kazemi S., Yaghooblou F., Siassi F., Rahimi Foroushani A., Ghavipour M., Koohdani F., Sotoudeh G. (2017). Cardamom supplementation improves inflammatory and oxidative stress biomarkers in hyperlipidemic, overweight, and obese pre-diabetic women: A randomized double-blind clinical trial. J. Sci. Food Agric..

[68] Peng J., Hu T., Li J., Du J., Zhu K., Cheng B., Li K. (2019). Shepherd’s purse polyphenols exert its anti-inflammatory and antioxidative effects associated with suppressing MAPK and NF-κB pathways and heme oxygenase-1 activation. Oxid. Med. Cell Longev..

[69] Shen Y., Zhang H., Cheng L., Wang L., Qian H., Qi X. (2016). In vitro and in vivo antioxidant activity of polyphenols extracted from black highland barley. Food Chem..

[70] Dharmawansa K.V.S., Hoskin D.W., Rupasinghe H.P.V. (2020). Chemopreventive effect of dietary anthocyanins against gastrointestinal cancers: A review of recent advances and perspectives. Int. J. Mol. Sci..

[71] Kępa M., Miklasińska-Majdanik M., Wojtyczka R.D., Idzik D., Korzeniowski K., Smoleń-Dzirba J., Wąsik T.J. (2018). Antimicrobial potential of caffeic acid against staphylococcus aureus clinical strains. Biomed. Res. Int..

[72] Kwak T.W., Park S.B., Kim H.-J., Jeong Y.I., Kang D.H. (2017). Anticancer activities of epigallocatechin-3-gallate against cholangiocarcinoma cells. Onco. Targets Ther..

[73] Malar D.S., Prasanth M.I., Brimson J.M., Sharika R., Sivamaruthi B.S., Chaiyasut C., Tencomnao T. (2020). Neuroprotective properties of green tea (Camellia sinensis) in Parkinson’s disease: A review. Molecules.

[74] Shen C.Y., Jiang J.G., Yang L., Wang D.W., Zhu W. (2017). Anti-ageing active ingredients from herbs and nutraceuticals used in traditional Chinese medicine: Pharmacological mechanisms and implications for drug discovery. Br. J. Pharmacol..

[75] Yu Y., Shen Q., Lai Y., Park S.Y., Ou X., Lin D., Jin M., Zhang W. (2018). Anti-inflammatory effects of curcumin in microglial cells. Front. Pharmacol..

[76] Berman A.Y., Motechin R.A., Wiesenfeld M.Y., Holz M.K. (2017). The therapeutic potential of resveratrol: A review of clinical trials. NPJ Precis. Oncol..

[77] Bielory L., Tabliago N.R.A. (2020). Flavonoid and cannabinoid impact on the ocular surface. Curr. Opin. Allergy Clin. Immunol..

[78] Chakrawarti L., Agrawal R., Dang S., Gupta S., Gabrani R. (2016). Therapeutic effects of EGCG: A patent review. Expert Opin. Ther. Pat..

[79] David A.V.A., Arulmoli R., Parasuraman S. (2016). Overviews of biological importance of quercetin: A bioactive flavonoid. Pharm. Rev..

[80] Kawabata K., Yoshioka Y., Terao J. (2019). Role of intestinal microbiota in the bioavailability and physiological functions of dietary polyphenols. Molecules.

[81] Santhakumar A.B., Battino M., Alvarez-Suarez J.M. (2018). Dietary polyphenols: Structures, bioavailability and protective effects against atherosclerosis. Food Chem. Toxicol..

[82] Teng H., Chen L. (2019). Polyphenols and bioavailability: An update. Crit. Rev. Food Sci. Nutr..

[83] Chojnacka K., Witek-Krowiak A., Skrzypczak D., Mikula K., Młynarz P. (2020). Phytochemicals containing biologically active polyphenols as an effective agent against Covid-19-inducing coronavirus. J. Funct. Foods.

[84] Hu J., Webster D., Cao J., Shao A. (2018). The safety of green tea and green tea extract consumption in adults—Results of a systematic review. Regul. Toxicol. Pharmacol..

[85] Abengózar-Vela A., Calonge M., Stern M.E., Gonzáles-García M.J., Enríquez-de-Salamanca A. (2015). Quercetin and resveratrol decrease the inflammatory and oxidative responses in human ocular surface epithelial cells. Biochem. Mol. Biol..

[86] Abu-Amero K.K., Kondkar A.A., Chalam K.V. (2016). Resveratrol and ophthalmic diseases. Nutrients.

[87] Bungau S., Abdel-Daim M.M., Tit D.M. (2019). Health benefits of polyphenols and carotenoids in age-elated eye diseases. Oxid. Med. Cell Longev..

[88] Eom Y., Song J.S., Kim H.M. (2016). Modified Haigis formula effective lens position equation for ciliary sulcus-implanted intraocular lenses. Am. J. Ophthalmol..

[89] Hayakawa S., Ohishi T., Miyoshi N., Oishi Y., Nakamura Y., Isemura M. (2020). Anti-cancer effects of green tea epigallocatchin-3-gallate and coffee chlorogenic acid. Molecules.

[90] Bimonte S., Cascella M. (2020). The potential roles of epigallocatechin-3-gallate in the treatment of ovarian cancer: Current state of knowledge. Drug Des. Devel. Ther..

[91] Cao T., Zhang X., Yang D., Wang Y.Q., Qiao Z.D., Huang J.M., Zhang P. (2018). Antioxidant effects of epigallocatechin-3-gallate on the aTC1-6 pancreatic alpha cell line. Biochem. Biophys. Res. Commun..

[92] Cui C.J., Jin J.L., Guo L.N., Liu G., Dong Q., Li J.J. (2020). Beneficial impact of epigallocatechingallate on LDL-C through PCSK9/LDLR pathway by blocking HNF1α and activating FoxO3a. J. Transl. Med..

[93] He F., Zhang Y., Chen S., Ye B., Chen J., Li C. (2018). Effect of EGCG on oxidative stress and Nrf2/HO-1 pathway in neurons exposed to oxygen-glucose deprivation/reperfusion. Zhong Nan Da Xue Xue Bao Yi Xue Ban.

[94] Mi Y., Liu X., Tian H. (2018). EGCG stimulates the recruitment of brite adipocytes, suppresses adipogenesis and counteracts TNF-α-triggered insulin resistance in adipocytes. Food Funct..

[95] Li K., Teng C., Min Q. (2020). Advanced nanovehicles-enabled delivery systems of epigallocatechin gallate for cancer therapy. Front. Chem..

[96] Shim W., Kim C.E., Lee M. (2019). Catechin solubilization by spontaneous hydrogen bonding with poly(ethylene glycol) for dry eye therapeutics. J. Control. Release.

[97] Cavet M.E., Harrington K.L., Vollmer T.R., Ward K.W., Zhang J.-Z. (2011). Anti-inflammatory and anti-oxidative effects of the green tea polyphenol epigallocatechine gallate in human corneal epithelial cells. Mol. Vis..

[98] Luo L.-J., Lai J.-Y. (2017). Epigallocatechin gallate-loaded gelatin-g-poly(N-Isopropylacrylamide) as a new ophthalmic pharmaceutical formulation for topical use in the treatment of dry eye syndrome. Sci. Rep..

[99] Stoddard A.R., Koetje L.R., Mitchell A.K., Schotanus M.P., Ubels J.L. (2013). Bioavailability of antioxidants applied to stratified human corneal epithelial cells. J. Ocul. Pharmacol. Ther..

[100] Lee H.S., Chauhan S.K., Okanobo A., Nallasamy N., Dana R. (2011). Therapeutic efficacy of topical epigallocatechin gallate (EGCG) in murine dry eye. Cornea.

[101] Tseng C.-L., Hung Y.-J., Chen Z.-Y., Fang H.-W., Chen K.-H. (2016). Synergistic effect of artificial tears containing epigallocatechin gallate and hyaluronic acid for the treatment of rabbits with dry eye syndrome. PloS ONE.

[102] Luo L.J., Nguyen D.D., Lai J.Y. (2020). Long-acting mucoadhesive thermogels for improving topical treatments of dry eye disease. Mater. Sci. Eng. C Mater. Biol. Appl..

[103] Huang H.Y., Wang M.C., Chen Z.Y., Chiu W.Y., Chen K.H., Lin I.C., Yang W.V., Wu C.C., Tseng C.L. (2018). Gelatin-epigallocatechin gallate nanoparticles with hyaluronic acid decoration as eye drops can treat rabbit dry-eye syndrome effectively via inflammatory relief. Int. J. Nanomed..

[104] Nejabat M., Reza S.A., Zadmehr M., Yasemi M., Sobhani Z. (2017). Efficacy of green tea extract for treatment of dry eye and meibomian gland dysfunction; a double-blind randomized controlled clinical trial study. J. Clin. Diagn. Res..

[105] Sedlak L., Wojnar W., Zych M., Wyględowska-Promieńska D., Mrukwa-Kominek E., Kaczmarczyk-Sedlak I. (2018). Effect of resveratrol, a dietary-derived polyphenol, on the oxidative stress and polyol pathway in the lens of rats with streptozotocin-induced diabetes. Nutrients.

[106] Meng T., Xiao D., Muhammed A., Deng J., Chen L., He J. (2021). Anti-inflammatory action and mechanisms of resveratrol. Molecules.

[107] Pagliaro B., Santolamazza C., Simonelli F., Rubattu S. (2015). Phytochemical compounds and protection from cardiovascular diseases: A state of the art. Biomed. Res. Int..

[108] Rauf A., Imran M., Butt M.S., Nadeem M., Peters D.G., Mubarak M.S. (2018). Resveratrol as an anti-cancer agent: A review. Crit. Rev. Food Sci. Nutr..

[109] Rahman M.H., Akter R., Bhattacharya T., Abdel-Daim M.M., Alkahtani S., Arafah M.W., Al-Johani N.S., Alhoshani N.M., Alkeraishan N., Alhenaky A. (2020). Resveratrol and neuroprotection: Impact and its therapeutic potential in Alzheimer’s disease. Front. Pharmacol..

[110] Shetty R., Subramani M., Murugeswari P., Anandula V.R., Matalia H., Jayadev C., Ghosh A., Das D. (2020). Resveratrol rescues human corneal epithelial cells cultured in hyperosmolar conditions: Potential for dry eye disease treatment. Cornea.

[111] Abengózar-Vela A., Schaumburg C.S., Stern M.E., Calonge M., Enríquez-de-Salamanca A., González-García M.J. (2019). Topical quercetin and resveratrol protect the ocular surface in experimental dry eye disease. Ocul. Immunol. Inflamm..

[112] D’Andrea G. (2015). Quercetin: A flavonol with multifaceted therapeutic applications?. Fitoterapia.

[113] Dos Santos M., Poletti P.T., Favero G., Stacchiotti A., Bonomini F., Montanari C.C., Bona S.R., Marroni N.P., Rezzani R., Veronese F.V. (2018). Protective effects of quercetin treatment in a pristane-induced mouse model of lupus nephritis. Autoimmunity.

[114] Marunaka Y., Marunaka R., Sun H. (2017). Actions of quercetin, a polyphenol, on blood pressure. Molecules.

[115] Shafabakhsh R., Asemi Z. (2019). Quercetin: A natural compound for ovarian cancer treatment. J. Ovarian Res..

[116] Li Y., Yao J., Han C., Yang J., Chaudhry M.T., Wang S., Liu H., Yin Y. (2016). Quercetin, inflammation and immunity. Nutrients.

[117] Dogan Z., Cetin A., Elibol E., Vardi N., Turkoz Y. (2019). Effects of ciprofloxacin and quercetin on fetal brain development: A biochemical and histopathological study. J. Mat. Fetal Neonatal. Med..

[118] Javadi F., Eghtesadi S., Ahmadzadeh A., Aryaeian N., Zabihiyeganeh M., Foroushani A.R., Jazayeri S. (2014). The effect of quercetin on plasma oxidative status, C-reactive protein and blood pressure in women with rheumatoid arthritis. Int. J. Prev. Med..

[119] McKay T.B., Karamichos D. (2017). Quercetin and the ocular surface: What we know and where we are going. Exp. Biol. Med..

[120] Oh H.N., Kim C.E., Lee J.H., Yang W.H. (2015). Effects of quercetin in a mouse model of experimental dry eye. Cornea.

[121] Dabeek W.M., Marra M.V. (2019). Dietary quercetin and kaempferol: Bioavailability and potential cardiovascular-related bioactivity in humans. Nutrients.

[122] Chen H.-C., Chen Z.-Y., Wang T.-J., Drew V.J., Tseng C.-L., Fang H.-W., Lin F.H. (2017). Herbal supplement in a buffer for dry eye syndrome treatment. Int. J. Mol. Sci..

[123] de Araújo F.F., de Paulo Farias D., Neri-Numa I.A., Pastore G.M. (2020). Polyphenols and their applications: An approach in food chemistry and innovation potential. Food Chem..

[124] Alam W., Khan H., Shah M.A., Cauli O., Saso L. (2020). Kaempferol as a dietary anti-inflammatory agent: Current therapeutic standing. Molecules.

[125] Barone E., Calabrese V., Mancuso C. (2009). Ferulic acid and its therapeutic potential as a hormetin for age-related diseases. Biogerontology.

[126] Chang K., Liu J., Jiang W., Zhang R., Zhang T., Liu B. (2020). Ferulic acid-ovalbumin protein nanoparticles: Structure and foaming behavior. Food Res. Int..

[127] Elansary H.O., Szopa A., Kubica P., Ekiert H., Al-Mana F.A., El-Shafei A.A. (2020). Polyphenols of *Frangula alnus* and *Peganum harmala* leaves and associated biological activities. Plants.

[128] Sahu R., Dua T.K., Das S., De Feo V., Dewanjee S. (2019). Wheat phenolics suppress doxorubicin-induced cardiotoxicity via inhibition of oxidative stress, MAP kinase activation, NF-κB pathway, PI3K/Akt/mTOR impairment, and cardiac apoptosis. Food Chem. Toxicol..

[129] Yao Y., Wang H., Xu F., Zhang Y., Li Z., Ju X., Wang L. (2020). Insoluble-bound polyphenols of adlay seed ameliorate H2O2-induced oxidative stress in HepG2 cells via Nrf2 signalling. Food Chem..

[130] Freyssin A., Page G., Fauconneau B., Rioux Bilan A. (2020). Natural stilbenes effects in animal models of Alzheimer’s disease. Neural. Regen. Res..

[131] Cassiano C., Eletto D., Tosco A., Riccio R., Monti M.C., Casapullo A. (2020). Determining the effect of pterostilbene on insulin secretion using chemoproteomics. Molecules.

[132] Peng R.M., Lin G.R., Ting Y., Hu J.Y. (2018). Oral delivery system enhanced the bioavailability of stilbenes: Resveratrol and pterostilbene. Biofactors.

[133] Ramezani G., Pourgheysari B., Shirzad H., Sourani Z. (2019). Pterostilbene increases Fas expression in T-lymphoblastic leukemia cell lines. Res. Pharm. Sci..

[134] Yao Y., Liu K., Zhao Y., Hu X., Wang M. (2018). Pterostilbene and 4’-Methoxyresveratrol inhibited lipopolysaccharide-induced inflammatory response in RAW264.7 macrophages. Molecules.

[135] La Spina M., Sansevero G., Biasutto L., Zoratti M., Peruzzo R., Berardi N., Sale A., Azzolini M. (2019). Pterostilbene improves cognitive performance in aged rats: An in vivo study. Cell Physiol. Biochem..

[136] Li J., Deng R., Hua X., Zhang L., Lu F., Coursey T.G., Pflugfelder S.C., Li D.Q. (2016). Blueberry component pterostilbene protects corneal epithelial cells from inflammation via anti-oxidative pPathway. Sci. Rep..

[137] Hu L., Hu Z., Yu Y., Ding X., Li K., Gong Q., Lin D., Dai M., Lu F., Li X. (2020). Preparation and characterization of a pterostilbene-peptide prodrug nanomedicine for the management of dry eye. Int. J. Pharm..

[138] Keshavarzi Z., Shakeri F., Barreto G.E., Bibak B., Sathyapalan T., Sahebkar A. (2019). Medicinal plants in traumatic brain injury: Neuroprotective mechanisms revisited. Biofactors.

[139] Panahi Y., Kianpour P., Mohtashami R., Jafari R., Simental-Mendía L.E., Sahebkar A. (2017). Efficacy and safety of phytosomal curcumin in non-alcoholic fatty liver disease: A randomized controlled trial. Drug Res..

[140] Peng K.-T., Chiang Y.-C., Huang T.-Y., Chen P.-C., Chang P.-J., Lee C.-W. (2019). Curcumin nanoparticles are a promising anti-bacterial and anti-inflammatory agent for treating periprosthetic joint infections. Int. J. Anomed..

[141] Shabbir U., Rubab M., Tyagi A., Oh D.H. (2020). Curcumin and its derivatives as theranostic agents in Alzheimer’s disease: The implication of nanotechnology. Int. J. Mol. Sci..

[142] Shabbir U., Rubab M., Daliri E.B., Chelliah R., Javed A., Oh D.H. (2021). Curcumin, quercetin, catechins and metabolic diseases: The role of gut microbiota. Nutrients.

[143] Stohs S.J., Chen O., Ray S.D., Ji J., Bucci L.R., Preuss H.G. (2020). Highly bioavailable forms of curcumin and promising avenues for curcumin-based research and application: A Review. Molecules.

[144] Chen M., Hu D.-N., Pan Z., Lu C.-W., Xue X.-Y., Aass Y. (2010). Curcumin protects against hyperosmocity-induced IL-1β elevation in human corneal epithelial cell via MAPK pathways. Exp. Eye Res..

[145] Muz O.E., Orhan C., Erten F., Tuzcu M., Ozercan I.H., Singh P., Morde A., Padigaru M., Rai D., Sahin K. (2020). A novel integrated active herbal formulation ameliorates dry eye syndrome by inhibiting inflammation and oxidative stress and enhancing glycosylated phosphoproteins in rats. Pharmaceuticals.

[146] Dobrzynska M., Napierala M., Florek E. (2020). Flavonoid nanoparticles: A promising approach for cancer therapy. Biomolecules.

[147] Xue B., Huang J., Zhang H., Li B., Xu M., Zhang Y., Xie M., Li X. (2020). Micronized curcumin fabricated by supercritical CO_2_ to improve antibacterial activity against *Pseudomonas aeruginosa*. Artif. Cells Nanomed. Biotechnol..

[148] Kawashima M., Ozawa Y., Shinmura K., Inaba T., Nakamura S., Kawakita T., Watanabe M., Tsubota K. (2013). Calorie restriction (CR) and CR mimetics for the prevention and treatment of age-related eye disorders. Exp. Gerontol..

